# The Effects of a Multidisciplinary Residential Program on the Risk Factors of Sarcopenic Obesity: An Open-Label Trial Study in a Cohort of Institutionalized Italian Adults with Obesity

**DOI:** 10.3390/nu17091511

**Published:** 2025-04-29

**Authors:** Mariangela Rondanelli, Clara Gasparri, Alessia Moroni, Elisa Genovese, Eugenio Marzio Valentini, Giorgia Leone, Simone Perna, Giuseppe Mazzola

**Affiliations:** 1 Department of Public Health, Experimental and Forensic Medicine, University of Pavia, 27100 Pavia, Italy; mariangela.rondanelli@unipv.it; 2 Endocrinology and Nutrition Unit, Azienda di Servizi alla Persona “Istituto Santa Margherita”, University of Pavia, 27100 Pavia, Italy; clara.gasparri01@universitadipavia.it (C.G.); alessia.moroni02@universitadipavia.it (A.M.); elisa.genovese01@universitadipavia.it (E.G.); eugeniomarzio.valentini01@universitadipavia.it (E.M.V.); giorgia.leone01@universitadipavia.it (G.L.); giuseppe.mazzola02@universitadipavia.it (G.M.); 3 Division of Human Nutrition, Department of Food, Environmental and Nutritional Sciences (DeFENS), Università degli Studi di Milano, 20133 Milan, Italy

**Keywords:** sarcopenic obesity, body composition, multidisciplinary residential program

## Abstract

**Background:** Sarcopenic obesity, characterized by excess fat and reduced muscle mass/function, is linked to chronic inflammation and metabolic dysfunction. **Methods:** This study aimed to evaluate the efficacy of a 2-month multidisciplinary residential program (MRP) on the clinical and functional outcomes associated with the risk of sarcopenia in 61 institutionalized Italian adults with obesity (mean age of 60; 36 women and 25 men; BMI ≥ 30 with metabolic comorbidities). The MRP included personalized nutrition, physical activity, and psychological support. Outcomes included anthropometric, biochemical, body composition, and physical performance measures (via Short Physical Performance Battery [SPPB]), with sarcopenia risk evaluated using EWGSOP2 criteria. **Results:** Post-intervention, significant improvements were observed in SPPB scores (+0.93 units, *p* < 0.001), weight (−6.4 kg), BMI (−2.45 kg/m^2^), fat mass (−3.9 kg), visceral adipose tissue (−314.2 g), and fat-free mass index (−285.54 g; all *p* < 0.01). Glycemic control improved, with reductions in fasting glucose (−16.4 mg/dL), HbA1c (−0.81%), insulin (−2.77 mcU/mL), and HOMA-IR (−0.95; *p* < 0.05). Lipid profiles also improved, including total cholesterol (−21.32 mg/dL), LDL (−12.10 mg/dL), and triglycerides (−39.07 mg/dL; all *p* < 0.001). **Conclusions:** The MRP effectively enhanced body composition, metabolic health, and physical function, underscoring its potential as a preferred strategy for managing sarcopenic obesity in institutional settings.

## 1. Introduction

Obesity represents a major global health concern, affecting both industrialized and developing countries with epidemic proportions. According to the 2022 European Regional Obesity Report published by the World Health Organization (WHO), 29% of European adults and nearly one in three children are either overweight or living with obesity [1]. In obesity, there is an increase in the accumulation of adipose tissue mainly at the visceral level [2]. The inflammatory process that occurs in visceral adipose tissue is closely related to insulin resistance since inflammation interferes with the normal function of insulin, contributing to impaired glucose regulation in the body. Furthermore, inflammation is often associated with abnormal tissue restructuring and can progress to fibrosis [2,3]. Obesity therefore causes physiological alterations that can cause chronic inflammation in the body, negatively influencing various systems such as the vascular, immune, metabolic, hormonal, and bone systems. This condition of chronic inflammation can inhibit muscle protein synthesis, favoring the development of sarcopenia, characterized by the progressive loss of muscle mass and strength. In particular, excess adipose tissue increases the production of cytokines, further aggravating muscle alterations [4]. Sarcopenia, initially defined in 2010 by the European Working Group on Sarcopenia in Older People (EWGSOP), was primarily characterized by the loss of skeletal muscle mass. In 2019, the revised EWGSOP2 consensus redefined sarcopenia as a progressive and generalized skeletal muscle disorder in which low muscle strength serves as the principal diagnostic criterion, with low muscle quantity or quality confirming the diagnosis and poor physical performance indicating severity [4]. This transition marked a paradigm shift, emphasizing muscle function over mass. A recent critical review further proposed the use of specific terms—myopenia (low muscle mass), dynapenia (low strength), and kratopenia (low muscle power)—to improve clinical specificity and phenotypic classification [5].

When sarcopenia coexists with obesity, the resulting clinical condition is termed sarcopenic obesity (SO). According to the ESPEN and EASO consensus statement (2022), SO is defined by the concurrent presence of excess adiposity and low muscle function (strength and/or mass), often associated with systemic inflammation, metabolic disturbances, and physical disability [6]. This phenotype is particularly concerning due to the synergistic negative effects of both components. The diagnosis of SO requires an integrated assessment that includes anthropometric measurements, functional tests, and body composition analysis [6]. Poor dietary intake—especially insufficient protein—and physical inactivity contribute to the progression of sarcopenic obesity, exacerbating the imbalance between lean and fat mass. This imbalance impairs muscle function both metabolically and mechanically, leading to reduced physical performance, lower exercise tolerance, and an increased risk of disability [6,7].

Given this background, the aim of this study was to evaluate the efficacy of a multidisciplinary residential program (MRP) in improving clinical and functional outcomes associated with the risk of sarcopenia in a cohort of Italian patients with obesity. The program targeted improvements in body composition, muscle strength, physical performance, and metabolic health, aligning with the multidimensional criteria used in contemporary definitions of sarcopenia and sarcopenic obesity.

## 2. Materials and Methods

### 2.1. Study Design and Population

This prospective, open-label observational cohort study was conducted at the Metabolic Rehabilitation Unit of the Azienda di Servizi alla Persona, Istituto Santa Margherita, University of Pavia (Pavia, Italy). Both participants and investigators were aware of the treatment protocol.

The study was approved by the Ethics Committee of the University of Pavia, and all participants provided written informed consent prior to enrollment.

Data were collected between 1 January 2021 and 1 September 2023 in accordance with the STROBE guidelines for observational studies and following the CONSORT reporting structure for transparency [8].

Inclusion criteria were as follows: age ≥ 18 years, BMI ≥ 30 kg/m^2^, and at least one metabolic comorbidity (e.g., type 2 diabetes, dyslipidemia, hypertension, or hyperuricemia). Exclusion criteria included the following: pregnancy or breastfeeding, active oncologic disease, severe psychiatric conditions (e.g., psychosis), severe cognitive impairment, or inability to participate in physical activity due to musculoskeletal or cardiovascular contraindications.

Participants were referred to the residential program by general practitioners, endocrinologists, or hospital-based specialists. A total of 102 individuals were initially screened for eligibility. Among these, 72 met the inclusion criteria and were invited to participate. Eleven individuals declined participation prior to the start of the program. The final sample included 61 participants (36 women and 25 men), all of whom completed the 8-week multidisciplinary residential program.

The multidisciplinary residential program (MRP) lasted approximately two months and was delivered in a fully supervised inpatient setting.

### 2.2. Multidimensional Residential Program Interventions

The multidisciplinary residential program (MRP) was designed as a comprehensive, hospital-based intervention integrating nutritional counseling, structured physical exercise, and psychological support. The rationale behind this approach is grounded in the complex, multifactorial pathophysiology of sarcopenic obesity, which involves metabolic, mechanical, and behavioral components. The program aimed to create a synergistic effect through simultaneous improvements in energy balance, muscle function, and cognitive emotional regulation. The three components of the MRP are detailed below.

#### 2.2.1. Nutritional Intervention

The nutritional intervention consisted of a low-energy mixed diet (50–55% carbohydrates, 25–30% lipids, 15–20% proteins) designed to provide a 500–600 kcal/day caloric deficit based on individual TEE, calculated from resting energy expenditure and activity factors. The diet adhered to American Diabetes Association guidelines [9] while ensuring a weekly weight loss of 0.5–1 kg (a low-risk approach [9]) and providing an adequate protein intake in line with official recommendations to meet the estimated protein requirements of individuals with obesity. Meals were tailored by registered dietitians to meet individual metabolic needs and food preferences. Weekly one-on-one dietary counseling sessions included goal setting, portion size education, and behavioral strategies for long-term adherence. Vitamin D supplementation was administered if 25OHD was <30 ng/mL.

#### 2.2.2. Physical Activity

The exercise regimen followed the World Health Organization’s recommendations [10] and consisted of a concurrent training protocol combining aerobic and strength-based exercises. Although no stratification was applied based on obesity severity, this approach was selected due to limited consensus regarding optimal exercise regimens in adults with severe obesity (BMI ≥ 40 kg/m^2^). Each participant engaged in a 60 min supervised exercise session five days per week, combining aerobic activities (e.g., treadmill walking or cycling) and resistance exercises (e.g., bodyweight and elastic band training). These were adapted to individual fitness levels and progressed weekly. The program also included a pedometer-based daily goal of ≥10,000 steps to encourage active behavior outside of structured sessions. This protocol was informed by previous evidence supporting combined training in improving physical function and metabolic health among individuals with obesity [11]. All sessions were tailored to individual capacity and were supervised on-site by licensed physiotherapists with expertise in obesity rehabilitation.

#### 2.2.3. Psychological Support

Psychological support was based on the enhanced cognitive behavioral therapy (CBT-E) framework [12], which is considered a first-line approach in the management of eating disorders and maladaptive dietary behaviors, and has demonstrated particular efficacy in the context of obesity, where emotional eating, cognitive distortions, and low self-efficacy often act as barriers to behavioral change. The intervention aimed to identify disordered eating patterns, promote cognitive restructuring, and provide psychoeducational tools to support behavioral change and adherence to nutritional recommendations. Participants attended weekly individual sessions with trained psychologists to address psychological barriers, reduce maladaptive thought patterns, and strengthen self-regulation strategies. In addition, multidisciplinary group meetings involving both psychologists and dietitians focused on skill-building for long-term weight maintenance and post-program relapse prevention.

### 2.3. Measured Outcomes

#### 2.3.1. Anthropometric Assessment

Anthropometric measurements, including body weight (recorded to 0.1 kg using a precision scale), waist circumference (measured at the midpoint between the iliac crest and the lowest rib), and hip circumference, were taken weekly throughout the 12-week multidisciplinary residential program (MRP). Weight was recorded to the nearest 0.1 kg using a calibrated digital scale, and waist circumference was measured at the midpoint between the iliac crest and the lowest rib. Participants were assessed while wearing light clothing and in accordance with standardized protocols [13].

#### 2.3.2. Body Composition via Dual-Energy X-Ray Absorptiometry (DXA)

Body composition was evaluated using a Lunar Prodigy DXA system (GE Medical Systems), including assessments of fat-free mass (FFM), fat mass, and visceral adipose tissue (VAT). The coefficients of variation were 0.89% for fat mass and 0.48% for FFM. The skeletal muscle index (SMI) was calculated as the sum of arm and leg FFM divided by height squared (kg/m^2^), while the fat-free mass index (FFMI) was calculated as the total FFM divided by height squared. FFM depletion was defined as FFMI below the 5th percentile for age- and sex-matched healthy controls [14]. VAT was estimated using a correction factor of 0.94 g/cm^3^, and subcutaneous abdominal fat was determined by subtracting VAT from android fat.

#### 2.3.3. Physical Performance

According to the updated criteria proposed by the European Working Group on Sarcopenia in Older People (EWGSOP2), probable sarcopenia is identified by low muscle strength—defined as handgrip strength below <27 kg in men and <16 kg in women, or the completion of a chair stand test taking longer than 15 s. The diagnosis is confirmed by low muscle mass, with cut-off values of appendicular skeletal muscle mass (ASM) < 20 kg for men and <15 kg for women, or ASM/height^2^ < 7.0 kg/m^2^ in men and <5.5 kg/m^2^ in women. Severe sarcopenia is diagnosed when poor physical performance is also present, defined by an SPPB score ≤ 8, gait speed ≤ 0.8 m/s, or a Timed Up and Go (TUG) test speed of ≥20 s [15].

*Handgrip Strength:* Handgrip strength was measured at baseline (t_0_) and post-intervention (t_1_) using a Jamar 5030 J1 hydraulic dynamometer (Sammons Preston Rolyan; 0.6 N accuracy). Participants were assessed while seated, with the elbow at the side and the arm flexed at a right angle, following standardized positioning.

*Short Physical Performance Battery (SPPB):* The Short Physical Performance Battery (SPPB) was used to assess lower extremity function at baseline (t_0_) and post-intervention (t_1_). The battery includes three components: a gait speed test (timed 4 m walk), a chair stand test (five consecutive rises from a seated position without arm support), and a balance assessment (side-by-side, semi-tandem, and tandem positions). Each component is scored from 0 to 4 based on performance time or ability to complete the task, with a maximum total score of 12 indicating optimal physical function [15].

#### 2.3.4. Biochemical Parameters

Blood samples were collected at baseline and post-intervention to assess nutritional status, lipid and glycemic profiles, inflammation, and metabolic health. Measurements included C-reactive protein (CRP), transferrin, Apo A1, and Apo B using immunoturbidimetry (Roche, Basel, Switzerland). Erythrocyte sedimentation rate (ESR) was assessed using the Westergren method (Diesse Analyzer). Electrolytes were analyzed by indirect ISE potentiometry (Abbott, Abbott Park, IL, USA), and ionized calcium was measured via selective electrode potentiometry. Insulin levels were quantified by electrochemiluminescence immunoassay (ECLIA, Roche). Glucose, AST, and ALT levels were determined by enzymatic UV assays (Abbott). Complete blood count (CBC) was determined with an automated differential blood cell counter. Insulin resistance was calculated using the HOMA index [16]. Additional parameters included serum iron, lipids, uric acid, creatinine, and total calcium, analyzed via enzymatic colorimetric assays (Abbott Laboratories).

### 2.4. Statistical Analysis

For continuous variables, paired t-tests were used to compare pre- and post-intervention measurements. For categorical variables, McNemar’s test was applied. While multiple outcome measures were assessed, no formal adjustment for multiple comparisons was implemented due to the exploratory nature of this observational study and the goal of hypothesis generation rather than strict statistical inference. This limitation is acknowledged, and *p*-values should be interpreted with appropriate caution regarding potential Type I errors. Statistical significance was set at *p* < 0.05.

Patient confidentiality was maintained through data anonymization, secure electronic storage on password-protected devices, and restricted access limited to authorized study personnel. All data handling procedures complied with institutional ethics guidelines and data protection regulations.

### 2.5. Ethical Considerations

The study protocol was conducted in accordance with the Declaration of Helsinki and approved by the Ethics Committee of the University of Pavia, Italy (approval number 1219/12062024). All participants provided written informed consent before enrollment. Patient confidentiality was ensured through anonymization of data, secure electronic storage, and restricted access to study personnel.

## 3. Results

A total of 61 patients with obesity completed the study (36 women and 25 men), with a mean age of 60.0 ± 13.5 years. Baseline characteristics for women and men, including anthropometric and functional parameters, are reported in Table 1.

The prevalence of diagnostic criteria associated with sarcopenia, based on EWGSOP2 thresholds, is summarized in Table 2 (baseline) and Table 3 (post-intervention). At baseline, no participant met the criteria for confirmed sarcopenia (i.e., low SMI), while 2 participants (3.28%) presented with low handgrip strength and 16 participants (26.23%) exhibited reduced physical performance in the chair stand test. These data support the preventive intent of the intervention, which targeted individuals at risk for sarcopenia rather than only those with established sarcopenic obesity.

As shown in Table 4, all the anthropometric and body composition variables significantly changed between pre and post recovery. In particular, weight, BMI, body circumference, fat mass, fat free mass, and VAT improved significantly (*p* < 0.001 or *p* < 0.01). Concerning physical performance, only SPPB values increased significantly after recovery (*p* < 0.001).

In Table 5, regarding blood parameters, as there was a significant improvement in the overall values between pre and post recovery, it is noteworthy to mention how such intervention enhanced glycaemia (*p* < 0.05), glycosylated hemoglobin (*p* < 0.01), insulinemia (*p* < 0.05), total cholesterol (*p* < 0.001), LDL (*p* < 0.001), and triglyceride (*p* < 0.001) levels.

## 4. Discussion

This prospective observational study demonstrates that a multidisciplinary residential program (MRP), delivered in a hospital-based setting with intensive supervision and standardization, can produce clinically relevant improvements in physical function, body composition, and metabolic parameters in individuals with obesity at risk of sarcopenia. Compared to prior outpatient or community-based interventions, our results suggest that the inpatient format may enhance adherence, optimize synergy among components, and facilitate more robust short-term functional gains [17,18,19]. The integrated design of the intervention, which combines nutritional counseling, structured physical activity, and psychological support, appears particularly suitable for addressing the multifactorial pathophysiology of sarcopenic obesity.

The observed improvements in SPPB scores, despite modest or non-significant changes in muscle mass and strength indices such as handgrip strength and SMI, suggest that functional adaptations may precede structural changes in muscle tissue. This phenomenon aligns with research indicating that neuromuscular efficiency and coordination may improve before detectable hypertrophy occurs, particularly in older adults. The non-significant changes in handgrip strength and SMI warrant consideration in the context of the relatively short intervention period, as structural adaptations in aging muscle typically require longer timeframes [20,21].

These findings support the concept that early functional gains may be mediated by improvements in neuromuscular activation, mitochondrial efficiency, and motor unit recruitment, especially in contexts of anabolic resistance. This is particularly relevant in sarcopenic obesity, where metabolic disturbances, chronic low-grade inflammation, and hormonal imbalances contribute to impaired muscle plasticity. Reduced visceral adipose tissue, which plays a key role in the inflammatory and endocrine crosstalk between adipose and muscle tissues, may also help restore a more anabolic and less catabolic environment. The downregulation of pro-inflammatory adipokines and improved insulin sensitivity may promote a metabolic context favorable to muscle function, even before substantial increases in muscle mass can occur [20,21].

The nutritional protocol adopted in this study, which included an energy-restricted diet with adequate protein intake, likely contributed to the preservation of lean mass during weight loss. Recent studies in the literature emphasize the importance of adeguate protein intake in preventing loss of muscle mass during caloric restriction in older adults with obesity [15]. Moreover, the structured physical activity program—combining aerobic and resistance elements—was designed to stimulate both cardiovascular adaptations and muscle activation, in line with recommendations for sarcopenia management [15].

The metabolic improvements observed following the intervention, including better glycemic control and lipid profiles, reflect the synergistic effect of body composition improvements and physical reconditioning. These effects are not merely secondary endpoints but represent core mechanisms in the prevention of sarcopenic trajectories, as insulin resistance and dyslipidemia directly impair anabolic signaling and increase muscle degradation pathways [20,21].

Finally, although improvements in micronutrient status such as vitamin B12 were not statistically significant, the trend toward normalization is clinically meaningful. Suboptimal B12 levels have been associated with impaired neuromuscular performance, and their correction may support broader rehabilitation goals.

### 4.1. Limitations

This study has several limitations. First, the lack of a control group limits causal inference, and the observed improvements may partially reflect regression to the mean, seasonal variation, or increased adherence due to the residential setting. Second, the intervention was conducted in a hospital-based environment, which limits generalizability to community or outpatient populations. Third, the relatively short duration of follow-up may not capture the full extent of muscular adaptations, particularly those related to structural remodeling. Fourth, the sample size was not determined through formal power analysis and may have limited the study’s ability to detect small effects or stratify by relevant subgroups such as sex. Additionally, no formal a priori power analysis was conducted to determine the sample size. Given the exploratory nature of the study and the scarcity of preliminary data on sarcopenia outcomes in obese institutionalized populations, the sample size of 61 participants was considered pragmatically feasible for a pilot observational design. However, this limitation may reduce the statistical power for detecting small effect sizes or for performing stratified analyses by sex or clinical subgroups. Finally, the analysis involved multiple statistical comparisons without correction for multiplicity, which may increase the risk of Type I error. Future randomized studies with longer follow-ups and mechanistic assessments are needed to confirm these findings and better delineate the biological pathways involved.

### 4.2. Future Clinical Application

Future clinical applications of this work could include the implementation of similar multidisciplinary programs in community or outpatient settings, with adaptations for scalability, accessibility, and cost-effectiveness. Such programs may play a central role in the structured management of sarcopenic obesity, particularly in settings where intensive, individualized interventions are not always feasible.

While randomized controlled trials (RCTs) remain the standard for evaluating intervention efficacy, the nature of multidisciplinary programs—encompassing behavioral, dietary, and physical activity components—requires open-label designs. In this context, pragmatic RCTs comparing MRPs with standard outpatient care represent a feasible and scientifically sound strategy to assess their effectiveness.

Although crossover designs are occasionally employed in nutrition research, they are less appropriate here due to the prolonged and multifactorial effects of residential interventions, which may not be reversible within acceptable washout periods.

Moreover, future studies should incorporate longer follow-up periods and include mechanistic assessments—such as inflammatory markers, muscle-specific biomarkers, and imaging data—to better understand the biological pathways underlying the clinical benefits of MRPs. Evaluating cost-effectiveness and patient adherence in real-world settings will also be essential for informing public health strategies and institutional policies.

## 5. Conclusions

In conclusion, this study highlights the clinical value of a multidisciplinary residential program in improving functional performance, body composition, and metabolic health in individuals with obesity who are at risk of sarcopenia. The findings support the notion that early functional gains can be achieved even in the absence of substantial structural muscle changes, particularly through improvements in neuromuscular coordination and metabolic efficiency. By targeting multiple domains—nutritional adequacy, physical reconditioning, and psychological support—within a supervised hospital-based setting, the intervention effectively addresses the complex pathophysiology of sarcopenic obesity. These results reinforce the importance of integrated, multicomponent strategies in mitigating the progression toward physical disability and metabolic deterioration in vulnerable populations. Future research should aim to replicate these findings in non-institutionalized settings, over longer durations, and with more detailed mechanistic exploration. Nevertheless, the present study contributes to the growing body of evidence advocating for structured and holistic approaches in the prevention and management of sarcopenic obesity.

## Figures and Tables

**Table 1:** Baseline characteristics of the sample.

Variable	Male (n = 36)	Female (n = 25)	Overall (n = 61)
Age (years)	60.5 ± 13.2	59.8 ± 14.1	60.0 ± 13.5
BMI (kg/m^2^)	41.1 ± 5.7	40.9 ± 6.2	41.0 ± 5.9
Waist (cm)	112.3 ± 9.8	110.5 ± 10.2	111.4 ± 10.0
Hip (cm)	135.2 ± 8.4	134.1 ± 8.9	134.7 ± 8.6
Handgrip (kg)	25.5 ± 8.2	24.3 ± 7.1	24.9 ± 7.7
SMI (kg/m^2^)	7.8 ± 1.2	7.5 ± 1.3	7.7 ± 1.2
SPPB Score	8.2 ± 2.1	7.9 ± 2.3	8.0 ± 2.2
Total Cholesterol (mg/dL)	185.0 ± 37.0	178.5 ± 34.5	182.6 ± 36.1
HDL (mg/dL)	46.0 ± 10.5	44.5 ± 9.8	45.9 ± 10.2
Triglycerides (mg/dL)	162.0 ± 72.0	158.0 ± 68.0	160.8 ± 70.0
LDL (mg/dL)	110.0 ± 33.0	106.0 ± 31.0	108.9 ± 32.0
Apo A (mg/dL)	140.0 ± 24.0	138.0 ± 22.0	139.2 ± 23.5
Apo B (mg/dL)	101.0 ± 26.0	98.0 ± 24.0	100.1 ± 25.0
CRP (mg/L)	3.2 ± 2.1	2.9 ± 1.8	3.0 ± 2.0
VAT (cm^2^)	150.0 ± 30.0	145.0 ± 28.0	147.5 ± 29.0
Fat-Free Mass (kg)	45.0 ± 8.0	44.5 ± 7.5	44.8 ± 7.8

Abbreviations: BMI, body mass index; SMI, skeletal muscle index; SPPB, short physical performance battery.

**Table 2:** Prevalence of sarcopenia-related criteria (risk factors for sarcopenia) at baseline (EWGSOP2).

	SMI	Handgrip Test	Chair Test	Handgrip Test AND Chair Test	Handgrip Test OR Chair Test
Cut-off	M < 7 kg/m^2^ F < 5.5 kg/m^2^	M < 27 kg F < 16 kg	>15 s	M < 27 kg F < 16 kg; >15 s	M < 27 kg F < 16 kg; >15 s
Men (n)	0	1	3	3	7
Women (n)	0	1	13	12	26
Total (n)	0	2	16	15	33
Total (%)	0%	3.3%	26.2%	24.6%	54.1%

Abbreviations: SMI, skeletal muscle index.

**Table 3:** Prevalence of sarcopenia-related criteria (risk factors for sarcopenia) after intervention (EWGSOP2).

	SMI	Handgrip Test	Chair Test	Handgrip Test AND Chair Test	Handgrip Test OR Chair Test
Cut-off	M < 7 kg/m^2^ F < 5.5 kg/m^2^	M < 27 kg F < 16 kg	>15 s	M < 27 kg F < 16 kg; >15 s	M < 27 kg F < 16 kg; >15 s
Men (n)	0	1	4	1	6
Women (n)	0	6	10	8	24
Total (n)	0	7	14	9	30
Total (%)	0%	11.5%	22.9%	14.75%	49.2%

Abbreviations: SMI, skeletal muscle index.

**Table 4:** Anthropometric parameters, body composition parameters, and physical function parameters at the beginning and at the end of recovery.

Variable	Pre (Mean ± SD)	Post (Mean ± SD)	Δ Change (CI95: Lower; Upper)	*p*-Value
Weight (kg)	106.5 ± 16.9	100.1 ± 16.0	−6.4 (−7.1; −5.7)	**0.001**
BMI (kg/m^2^)	41.1 ± 5.7	38.7 ± 5.6	−2.5 (−2.7; −2.2)	**0.001**
Arm circumference (cm)	36.8 ± 4.7	35.4 ± 3.8	−1.4 (−2.4; −0.4)	**0.007**
Calf circumference (cm)	42.1 ± 4.5	41.1 ± 3.5	−1.1 (−1.9; −0.2)	**0.017**
Waist circumference (cm)	125.9 ± 10.6	120.2 ± 10.8	−5.7 (−6.4; −4.9)	**0.001**
Hips circumference (cm)	127.6 ± 12.5	123.9 ± 12.4	−3.7 (−4.5; −3.0)	**0.001**
Total mass (kg)	106.5 ± 16.9	100.1 ± 16.0	−6.4 (−7.1; −5.7)	**0.001**
Fat mass (g)	49,411.1 ± 10,603.7	45,504.5 ± 10,428.6	−3906.7 (−4574.5; −3238.9)	**0.001**
Fat mass (%)	48.6 ± 6.9	46.7 ± 7.3	−1.9 (−2.4; −1.4)	**0.001**
Fat free mass (g)	51,976.3 ± 10,394.0	51,174.9 ± 9799.7	−801.5 (−1335.3; −267.6)	**0.004**
FFMI	19,889.8 ± 2279.4	19,604.3 ± 2192.7	−285.5 (−487.3; −83.8)	**0.006**
VAT (g)	2701.3 ± 1272.2	2387.1 ± 1098.9	−314.2 (−452.1; −176.3)	**0.001**
Handgrip (kg)	25.5 ± 12.1	26.1 ± 11.5	1.1 (−0.3; 2.5)	0.121
SMI	9.2 ± 1.2	9.1 ± 11.2	−0.1 (−0.3; 0.1)	0.248
SPPB (score)	8.5 ± 2.7	9.4 ± 2.3	0.9 (0.6; 1.3)	**0.0001**

Abbreviations. BMI, body mass index; FFMI, fat free mass index; VAT, visceral adipose tissue; SMI, skeletal muscle index; SPPB, short physical performance battery. In bold: values with *p* < 0.05.

**Table 5:** Blood chemistry parameters at the beginning and end of recovery.

Variable	Pre (Mean ± SD)	Post (Mean ± SD)	Δ Change (95% CI)	*p*-Value
Albumin (%)	58.9 ± 4.1	59.1 ± 4.3	+0.2 (−0.5; 0.9)	0.561
Albumin (g/dL)	4.0 ± 0.3	3.9 ± 0.4	−0.1 (−0.2; 0.0)	0.094
Alpha-1 globulin (%)	4.1 ± 0.6	4.0 ± 0.6	−0.1 (−0.4; 0.3)	0.734
Alpha-2 globulin (%)	10.3 ± 1.8	10.4 ± 1.8	+0.1 (0.0; 0.3)	**0.022**
ALT (IU/L)	33.6 ± 23.0	33.1 ± 27.4	−0.5 (−6.6; 5.6)	0.856
Amylase (U/L)	49.9 ± 19.6	45.2 ± 20.0	−4.8 (−7.2; −2.3)	**0.001**
Apo A (mg/dL)	139.2 ± 23.7	120.5 ± 20.0	−18.7 (−23.6; −13.8)	**0.001**
Apo B (mg/dL)	100.1 ± 26.0	84.7 ± 21.9	−15.3 (−20.5; −10.1)	**0.001**
AST (IU/L)	22.3 ± 11.1	20.4 ± 11.3	−1.9 (−4.8; 0.9)	0.182
Beta globulin (%)	12.0 ± 1.6	11.8 ± 1.8	−0.2 (−0.5; 0.1)	0.188
BUN (mg/dL)	39.8 ± 14.1	40.2 ± 16.7	+0.5 (−1.8; 2.8)	0.669
Calcium (mg/dL)	9.2 ± 0.6	9.4 ± 0.5	+0.1 (−0.0; 0.3)	0.119
Chloride (Cl, mmol/L)	102.7 ± 2.5	103.4 ± 3.7	+0.6 (0.4; 1.7)	0.242
CRP (mg/dL)	0.6 ± 0.6	0.5 ± 0.5	−0.1 (−0.3; 0.1)	0.144
Creatinine (mg/dL)	0.9 ± 0.3	0.9 ± 0.3	+0.0 (−0.0; 0.1)	0.402
ESR (mm/h)	27.5 ± 20.4	26.1 ± 15.0	−1.4 (−7.2; 4.4)	0.635
Folate (ng/mL)	7.5 ± 4.2	7.4 ± 13.1	−0.1 (−3.0; 2.8)	0.919
Gamma globulin (%)	14.8 ± 2.9	14.9 ± 2.8	+0.1 (−0.2; 0.4)	0.517
GGT (U/L)	32.5 ± 20.3	22.0 ± 14.9	−10.5 (−15.4; −5.6)	**0.001**
Glycaemia (mg/dL)	108.5 ± 52.3	92.1 ± 14.4	−16.4 (−31.0; −1.8)	**0.028**
HbA1c (%)	7.1 ± 1.6	6.3 ± 0.9	−0.8 (−1.3; −0.3)	**0.004**
Hematocrit (%)	41.2 ± 3.6	40.9 ± 3.5	−0.3 (−0.9; 0.3)	0.359
Hemoglobin (g/dL)	13.7 ± 1.4	13.6 ± 1.3	−0.2 (−0.3; 0.0)	0.130
HDL (mg/dL)	45.9 ± 10.2	40.1 ± 8.4	−5.8 (−7.4; −4.2)	**0.001**
Homocysteine (μmol/L)	16.1 ± 5.0	14.7 ± 5.9	−1.3 (−5.5; 2.8)	0.419
HOMA-IR	4.0 ± 2.8	3.1 ± 1.9	−1.0 (−1.8; −0.2)	**0.022**
Insulin (mcU/mL)	15.5 ± 8.7	12.8 ± 7.9	−2.8 (−5.1; −0.4)	**0.023**
Iron (mcg/L)	90.0 ± 33.7	107.1 ± 23.7	−17.1 (−25.5; −8.7)	**0.001**
Leukocytes (k/μL)	7.1 ± 2.0	6.5 ± 2.0	−0.7 (−1.0; −0.3)	**0.001**
Lipase (U/L)	23.0 ± 11.6	27.7 ± 14.9	+4.6 (2.0; 7.3)	**0.001**
Lymphocytes (n)	2.4 ± 0.9	2.3 ± 0.9	−0.1 (−0.2; 0.1)	0.488
Lymphocytes (%)	32.5 ± 7.1	35.8 ± 8.8	+3.3 (1.7; 4.9)	**0.001**
Mean corpuscular volume (fL)	87.9 ± 4.8	88.2 ± 4.4	+0.2 (−0.3; 0.8)	0.396
Phosphatase (U/L)	65.5 ± 17.7	62.2 ± 18.0	−3.2 (−6.7; 0.5)	0.086
Platelets (k/μL)	247.8 ± 247.8	236.6 ± 55.8	−11.2 (−21.4; −1.0)	**0.032**
Potassium (K, mmol/L)	4.4 ± 0.4	4.3 ± 0.5	−0.1 (−0.2; 0.0)	0.144
Prealbumin (mg/dL)	25.3 ± 4.9	23.5 ± 4.8	−1.8 (−2.9; −0.7)	**0.002**
Sodium (Na, mmol/L)	139.0 ± 2.1	140.3 ± 2.2	+1.3 (0.6; 2.1)	**0.001**
Total bilirubin (mg/dL)	0.7 ± 0.3	0.6 ± 0.3	−0.1 (−0.2; −0.1)	**0.001**
Total cholesterol (mg/dL)	182.6 ± 36.1	161.3 ± 35.7	−21.3 (−30.7; −12.0)	**0.001**
Total proteins (g/dL)	6.8 ± 0.5	6.6 ± 0.5	−0.2 (−0.3; −0.0)	**0.008**
Transferrin (mg/dL)	258.2 ± 43.7	241.6 ± 43.3	−16.6 (−27.9; −5.3)	**0.006**
Triglycerides (mg/dL)	160.8 ± 71.3	121.7 ± 39.4	−39.1 (−53.3; −24.9)	**0.001**
Uricemia (mg/dL)	6.0 ± 1.7	6.2 ± 1.9	+0.2 (−0.1; 0.5)	0.108
Vitamin B12 (pg/mL)	437.3 ± 184.5	506.1 ± 207.6	+68.8 (−122.4; 259.9)	0.335
Vitamin D (ng/mL)	25.0 ± 11.9	26.9 ± 13.7	+1.9 (−3.0; 6.8)	0.338

In bold: values with *p* < 0.05.

## Data Availability

The raw data supporting the conclusions of this article will be made available by the authors upon request. The data are not publicly available due to privacy reasons.
